# Fostering gerontology students’ competence in Interprofessional collaborative practice

**DOI:** 10.1186/s12909-020-02273-4

**Published:** 2020-10-27

**Authors:** Janita Pak Chun Chau, Suzanne Hoi Shan Lo, Vivian Wing Yan Lee, Wai Ming Yiu, Helen Chung Yan Chiang, David R. Thompson, Alexander Yuk Lun Lau

**Affiliations:** 1 The Nethersole School of Nursing, Faculty of Medicine, The Chinese University of Hong Kong, Shatin, N.T., Hong Kong SAR; 2 Centre for Learning Enhancement And Research, The Chinese University of Hong Kong, Shatin, N.T., Hong Kong SAR; 3 School of Nursing and Midwifery, Queen’s University Belfast, Belfast, UK; 4 Department of Medicine and Therapeutics, Faculty of Medicine, The Chinese University of Hong Kong, Shatin, N.T., Hong Kong SAR

**Keywords:** Interprofessional relations, Geriatrics, Education

## Abstract

**Background:**

Interprofessional collaborative practice (IPCP) is increasingly recognised as being crucial for the provision of holistic care and optimising health outcomes among older adults, many with multiple complex health problems. However, little is known about the challenges of facilitating this in practice. Therefore, this study explores these issues from the perspective of different healthcare professionals and how this might inform interprofessional education curricula.

**Methods:**

Sixteen different healthcare professionals working in a variety of aged care (acute, rehabilitative and community) settings were invited to participate in individual semi-structured in-depth interviews designed to: (i) explore the meaning of IPCP; (ii) explore the facilitators of and barriers to IPCP; and (iii) examine the opportunities and challenges in interprofessional gerontological education. All interviews were tape-recorded and transcribed verbatim with thematic analysis conducted by two independent researchers.

**Results:**

Three major themes emerged from the interviews: the need for IPCP; role preparedness, scope and liability; and strategies for interprofessional education. Respondents shared a common belief that IPCP improves the quality of life of older adults in both hospital and community settings by improving person-centred coordinated care and decision making in care planning. However, respondents perceived major barriers to IPCP to be lack of knowledge about healthcare professionals’ scope of practice, lack of training in interprofessional collaboration, professional culture and stereotypes, and liability issues. Suggested approaches to overcome these barriers included innovative teaching and learning approaches, engaging students early on in the curriculum of health professional degree programmes, and enhancing collaborative effective communication in health and social care settings.

**Conclusions:**

It is anticipated that these findings will be used to inform the development of a new interprofessional gerontological education curriculum that aims to enhance students’ competence in IPCP.

## Background

Interprofessional collaborative practice (IPCP) is an aspirational holistic approach to health care that accelerates the provision of high-quality care and comprehensive health services by a multidisciplinary team that works with patients and clients, their families, and their formal and informal caregivers across various healthcare and community settings [1]. An effective interprofessional team is characterised by the knowledge of each member about the roles and responsibilities of various professions and respecting and valuing each professional’s unique contributions to managing care [2].

For older adults, many with chronic conditions, IPCP aims to ensure the provision of truly holistic, person-centred care, as well as support to their families and caregivers. A systematic review of 37 randomised controlled trials reported that IPCP for community-dwelling older adults potentially improves care processes and reduces hospital or home care use [3]. Older adults recovering from acute conditions require both physical and psychosocial preparedness for discharge, and successful implementation of IPCP during this stage is crucial [4]. Sustainable primary care is accomplished by reducing health-related risk factors, which consequently decreases health service utilisation and yields higher quality outcomes [5]. Thus, for example, appears to be effective in addressing lifestyle risk factors such as promoting long-term weight loss [6].

The Bachelor of Science in Gerontology (BScG) programme is the first government-funded of its type in Hong Kong that addresses population ageing and a shortage of the aged care workforce. Graduates of this programme are expected to collaborate with multiple healthcare professionals to optimise the health and wellbeing of older adults in the community, residential or hospital settings. The programme endeavours to enrich the curriculum with innovative features designed to develop students’ competence in IPCP and prepare them to take an active role in IPCP after their graduation. Given the dearth of studies examining IPCP in the provision of aged care and of interprofessional gerontological education, we conducted a study that aimed to (i) explore the meaning of IPCP among aged care professionals; (ii) explore the facilitators of and barriers to IPCP; and (iii) examine the opportunities and challenges in interprofessional gerontological education.

## Methods

A qualitative study was conducted using individual semi-structured in-depth interviews with 16 aged care provider from a variety of professional backgrounds (two registered nurses, four social workers, two physicians, two pharmacists, two physiotherapists, two occupational therapists, and two BScG graduates), who had been involved in interdisciplinary team care for older adults for at least four years. After obtaining a written informed consent, the respondents were invited by a research assistant to share their views about IPCP in aged care using a semi-structured interview guide. The respondents were also invited to share their perceived meaning of IPCP; their experiences of, facilitators of and barriers to IPCP in aged care; strategies for enhancing IPCP, and opportunities and challenges in providing interprofessional gerontological education. All interviews were tape-recorded and transcribed verbatim. We adopted a methodological framework of member checking, debriefing, audit trail, and long engagement to guide the data analysis [7]. The themes identified from the transcripts and the corresponding supporting quotes were confirmed with relevant participants. Two investigators (JPCC and SHSL) regularly provided concrete reflective comments on the transcripts, codes, and findings. Regular debriefings were conducted to resolve the emergent concerns or issues [8]. The study was approved by the Survey and Behavioural Research Ethics Committee at the University. A consent form was signed by all respondents.

## Results

Each interview lasted from 45 to 90 min. Of the 16 respondents, nine (56.3%) were male, and 11 (68.8%) had over 10 years’ related work experience. Eleven (68.8%) had completed a masters or doctoral degree, four (25%) a bachelor degree and 1 (6.3%) an associate degree.

### Theme 1: need for IPCP

#### Enhanced holistic and person-centred coordinated care

The respondents consistently mentioned that IPCP represented the key to provision of holistic and person-centred care across acute and rehabilitation hospitals and community and residential care settings. They regarded IPCP as essential to boosting the sustainability of health care, with its importance being most evident in the care of older adults with complex health needs during the different phases of their chronic illness trajectory.

*‘Older adults’ health problems may change when they are in hospital and then return home…one health profession may be in charge of their care,* e.g. *during acute management of the disease, yet another profession’s expertise may be needed to provide holistic care, especially during discharge planning and long-term care planning.’ (Physician A).*

The importance of IPCP in enhancing person-centred coordinated care was illustrated by a pharmacist recounting an experience of fall risk evaluation and management for an older adult:

*‘Physicians will discuss whether the client requires a surgery to prevent falls, pharmacists will offer advice on what medications could reduce the risk of falls, and nurses will assess the client’s physical fitness and identify measures to prevent falls.’(Pharmacist A).*

A collaborative approach to care, drawing on the combined expertise of various professionals, that address older adults’ health and social problems holistically, was evident:

*‘Early discharge reduces complications associated with an extended hospital stay.… Careful and robust discharge planning is necessary and should be considered from multiple perspectives, i.e. after input from different health professionals.’(Physician B).*

*‘Social workers and aged care providers can help to address family dynamics-associated problems and financial distress that the clients might be experiencing. They are experts in mobilising resources that are available in society.’(BScG graduate A).*

A social worker argued that IPCP can be applied to many aspects of aged care and is particularly useful in managing older adults with chronic conditions and planning lifestyle changes.

*‘A client’s health problems can be complex, and you need different health professions’ expertise to address it.… Clients are often unaware of the association between suboptimal lifestyle and health, for instance, their diets may be unbalanced and they may be receiving inadequate care. Health professionals can identify the root causes of their health problems.’ (Nurse A).*

Respondents elaborated on the advantages of IPCP in terms of time, resources and communication which contributed to enhancing person-centred coordinated aged care.

*‘(IPCP) saves time and enables better use of resources, for example, no need to repeat assessment with the clients for diagnosis or monitoring’ (Physician 1).*

*‘(IPCP) promotes communication among different healthcare professions … it facilitates understanding of patients’ needs and referrals’ (Social Worker 1).*

A physiotherapist noted that inter-disciplinary health consultation services are offered for newly discharged older adults in the hospital. The team managing the care of clients in the geriatric day hospital communicates with the healthcare team in the hospital about the clients’ health condition and the challenges they may encounter after discharge.

*‘The services help the clients to achieve better control of their conditions and thus reduce the likelihood of re-admission.’ (Physiotherapist A).*

#### Shared decision making in care planning

Respondents mentioned that each professional shared their own expertise to promote holistic person-centred care. To achieve this, each member of the team needs to consult and coordinate with other team members for their expertise in developing a holistic care plan for clients.

*‘In healthcare, there are always areas that one does not know and requires input from others.… During case conferences, different professionals came together to discuss the best interventions for clients.… As different health professionals may visit the clients on separate occasions, a more holistic picture of the clients’ conditions can be drawn when health professionals exchange their information during a case conference.’ (Pharmacist B).*

*‘A pharmacist can advise on a better alternative to certain medication, the shelf life of medications, or whether a drug is compatible with another. Nurses invest a substantial amount of time in taking care of their clients and thus know more about their conditions. Social workers can advise on financial support for specific measures to be taken. All of this can help to set achievable goals for the clients via consensus among the professionals.’ (Physician B).*

During the process of shared decision making, respondents said the physician usually assumed the role of leading the discussion or case conferences.

*‘Each professional represents their own profession’s views on the clients’ care, but still need a person to overlook the case, and often the doctor will be the one, who knows more about the medical management …’ (Physician 2).*

However, respondents highlighted that the physician’s role was shifting more towards that of a ‘facilitator’. This was echoed by some respondents’ comments that there should be a person who led the discussions and this person would be the professional who was accountable for the key issues or health complaints of the older adult.

*‘In the community settings, nurses or persons who know more about the older adults usually take up the role of a case manager’ (Social Worker 2).*

### Theme 2: role preparedness, scope and liability

#### Knowledge about health professions’ scope of practice

Competencies in providing quality and safe care for older adults, interprofessional communication and relations, and teamwork are necessary for the successful IPCP. A physician stated that the successful implementation of IPCP depends on each professional’s understanding of self-strength and weakness.

*‘The professionals should know when they should seek advice from others and when to give suggestions to improve the clients’ outcomes.’ (Social worker A).*

Several respondents highlighted that adequate knowledge about other professional’s roles and responsibilities are crucial for building trust within an interprofessional team.

*‘Inexperienced clinicians may still be exploring their profession and may face difficulties in understanding the functions of other professionals and expressing their views. We need to understand the jargons, philosophies, and expertise of other health professionals to communicate effectively with them.’ (BScG graduate B).*

A social worker said that when discussing a case with nurses, he has to tune himself to the nurse’s ‘language’, such as talking about the clients’ diet and elimination, vital signs, blood pressure, and heart rate. These skills are difficult to acquire.

*‘Communication is crucial. Different professions may have different professional boundaries that need to be respected, or the collaborative relationship may be ruined.’ (Social worker B).*

Each professional presented his/her own professional views on client care, but several highlighted the need for a single person to supervise the case. In this regard, the care coordinator serves as a communication hub and facilitates teamwork by interacting with the clients and interdisciplinary care teams. A BScG graduate said they often take up this role in community settings and perform comprehensive assessment and initiate referrals to ensure the best possible care for the client. They are expected to be equipped with essential medical knowledge and knowledge of other professional’s roles.

#### Lack of training in interprofessional collaboration

All respondents agreed that the success of IPCP depends on effective and continuous communication among the team members. Conflicts may arise when a member gives opinions that are too offensive, as stated by one respondent. When a member becomes too dominant, the discussion will also be obstructed. Negotiation skills and a mindset for compromise are necessary for IPCP to ensure that a mutually-agreed decision is reached by all team members.

*‘Non-governmental organisations often have to purchase the services provided by nurses, physiotherapists, or occupational therapists in a pay-as-you-go approach. Every time a brand-new team is formed with new staff, it takes time for everyone to adapt and familiarise with their teammates. To avoid misunderstanding, one has to be tactful when delivering messages. Communicating in a concise manner and avoiding medical jargons would be desired for ensuring that things run smoothly.’ (BScG graduate A).*

*‘Those who have worked in community settings often have to deal with clients on their own or with minimal supervision. They have to make decisions even when IPCP is established as other health professionals are unavailable nearby for answering queries. The currently available training for facilitating the collaboration of professionals in such a dynamic team is inadequate.’ (Social worker C).*

A physiotherapist commented that the lack of health professionals’ willingness to create more opportunities to IPCP in community settings makes the situation worse.

*‘The colleagues who propose or initiate new IPCP services often have to rely on themselves for data searching and planning to convince their supervisors. They have to be savvy about finding a good time to introduce their ideas to the concerned parties. They have to make acquaintance with many parties before implementing the IPCP initiatives. These abilities cannot be acquired in a short time.’ (Physiotherapist B).*

#### Perceived roles and stereotypes

Owing to differences in power and status among aged care professionals, the general public tends to have more trust in physicians than in other health professionals. The authoritative image of physicians is regarded as a deep-rooted stereotype among Chinese.

*‘Health professionals other than physicians may feel discouraged as they are given little respect and thus become more reluctant to assume a role with broader responsibilities. The obligation for making the overall decision is often imposed on physicians, who are stereotypically perceived as the gatekeepers in hospitals.’ (Occupational therapist A).*

#### Liability issues

Several respondents commented that the acceptance of IPCP varies among health professionals. The involved parties often raise concerns regarding accountability issues.

*‘Liability issues are important.… People may be afraid of what they are responsible for, and if it is not a concern of my profession, it is better to leave it to others.’ (Physiotherapist B).*

One respondent expressed concerns that IPCP could be abused, e.g. a party may try to evade its responsibilities and impose them on others. He also stated that physicians and nurses often have to deal with emergency or life-and-death issues, and these conditions cannot be handled by allied health professionals. Therefore, IPCP serves limited functions in some settings.

The degree of accuracy of the provided information is determined by the team members’ competencies, and it may significantly affect the team’s performance.

*‘The teammate has to ensure that the information provided to other parties is correct. Once a wrong message is delivered, significant effort is required to rectify it as the message may have already been passed to multiple parties… Besides, a member may hesitate when referring a client to or requesting help from another professional as he/she may not be sure whether this could address the issue. These considerations and experiences could have a negative impact on initiating new collaboration with other health professionals.’ (Social worker D).*

### Theme 3: strategies for interprofessional education

#### Using innovative simulation-based teaching and learning strategies

Attaining teamwork synergy requires individual members learning to work separately as well as collaboratively. Respondents consistently mentioned that students from different health professions learn separately. They seldom have opportunities to study a course together or work collaboratively in practice.

*‘Innovative teaching strategies, such as role playing or simulation, would help students understand the implicit culture and unspoken rules of the workplace. This would help students to get involved in IPCP upon graduation.’ (Occupational therapist B).*

*‘Although it is good to introduce innovative teaching and learning strategies to let students know what working with different health professionals is like, there are logistics problems, e.g. ratio of teachers to students in different health professions, teaching room size, and levels of difficulty of the case scenarios.’ (Pharmacist B).*

#### Engaging students earlier in the curriculum

Learning is a process in which each health professional acquires the skills and mindset to participate in IPCP. Several respondents said, *‘It would be better to learn the right concept at the very beginning rather than rectifying it later.’* They stated that understanding the practice of other professionals via IPE during undergraduate study can prevent the formation of wrong perceptions or stereotypes about healthcare professions.

*‘Many gerontology students were eager to contribute to team meetings and case conferences during their community practicum. However, some reported inadequate confidence in coordinating care and insufficient ability to serve as advocates of their clients and interact with the team.… More input about high-impact communication skills and leadership skills in care coordination could help enhance their skills in applying interdisciplinary care principles in planning health and social care for older adults and their caregivers.’ (BScG graduate B).*

#### Enhancing interpersonal skills and effective communication

Interprofessional communication skills are essential for maintaining team dynamics.

*‘Interpersonal skills are essential to foster IPCP. Interpersonal skills such as being humble, showing courtesy, and expressing gratitude are facilitators for collaboration.’ (Nurse B).*

*‘An open mind and flexibility are vital as IPCP requires coordination with all involved parties.’ (BScG graduate B).*

*‘The use of social skills, such as using simple messages and showing respect to others, would allow all parties to build mutual understanding and lead to sustainable collaboration.… One may use euphemism and express opinions but should avoid being unpleasant or offensive to others. One should also avoid explicitly pointing out others’ wrongdoings as it may create hard feelings in others.’ (Physician B).*

*‘Communication can be as simple and direct as sending text or voice messages via smartphones in daily practice … We are all busy, but short messages could update each other instantly … Sometimes, adding a sense of humour in correspondences is powerful in getting each other closer.’ (Physician A).*

## Discussion

The findings from this study highlight the meaning and importance of IPCP as perceived by a variety of health professionals and offer pointers to how interprofessional gerontological education can be enhanced. Health professionals were positive about the principles and practise of IPCP as integral to the provision of care for older adults with common, complex and interrelated health conditions in acute, rehabilitative, residential, and community settings. The integration of expertise afforded by the different health professionals through IPCP was perceived by respondents as integral to achieving holistic person-centred coordinated care for older adults. The benefits of IPCP identified by the respondents included reductions in fall risk, length of hospital stay, and readmission rates, and enhancement of continuity of care, transitional care and end-of-life care planning. The findings are consistent with those of a systematic review [9], which reported significant positive associations between IPCP for elderly clients and professional and client satisfaction, quality of care, safety, and independence in daily activities.

The respondents in this study emphasised that IPCP is characterised by shared decision making—a collaborative process in which a healthcare decision is made with agreement between the client, his/her family, and the interprofessional team [10]. The findings suggest that sharing of assessment data among the interprofessional team members enhanced their understanding of the older adults’ health condition, allowing them to identify the optimal healthcare options. It also enabled more efficient use of the professionals’ and clients’ time as well as healthcare resources by minimising unnecessary repetition of some assessments or diagnostic tests. With increasing evidence suggesting the importance of self-management in managing common chronic diseases among older adults, the interprofessional team’s competence to comprehensively assess and identify the needs of older adults and their families is crucial to health-related decision making [10].

The major challenges of implementing IPCP identified in this study were similar to those reported in other studies [11, 12], including the team members’ experience, use of language, and interprofessional hierarchies. To address these challenges, our respondents consistently emphasised that the key step to promote IPCP was to understand the roles and expertise of each health professional member,: an essential skill for successful IPCP [13]. Each professional needs to recognise and, more importantly, respect the roles, responsibilities, competencies, and limitations of other professionals in relation to one’s own in order to achieve a common goal [13]. Lack of such understanding may cause ambiguity about one’s identities, values, and roles as well as tension about professional boundaries [13, 14]. Some respondents elaborated that each member in the interprofessional team could be the ‘manager’ of an older adult’s care plan. Though common practice, it is not necessary for a physician to always assume the stereotypical authoritative role, but rather the professional who can best manage the older adult’s care priorities and health condition at that time. Importantly, regardless of the professional who is in charge, all professionals have to be accountable for their own practice [10].

Effective communication skills were highlighted by our respondents as integral to IPCP because they help moderate the team members’ behaviours and maintain the team dynamics. Care coordination and teamwork, which require good communication skills, are crucial for successful care and health outcomes of high-need older adults [15]. A systematic review of 16 qualitative studies reported the importance of effective communication in clarifying and negotiating roles and developing cooperative relationships among interprofessional team members [11]. Some respondents in our study reported that direct communications during daily practice, such as text or voice messages via smartphones, facilitated sharing updated information about clients and strengthening of social interaction and bonding among team members. This concurs with a systematic review of six studies concurred that the use of information and communication technology facilitates IPCP [10].

All respondents suggested that interprofessional education offered early in undergraduate degrees is an effective approach to promote IPCP, and this supports findings that integrating interprofessional education early in the curriculum helped students gain insights into the merits of collaboration and teamwork and learn strategies to overcome possible difficulties during IPCP [16]. A systematic review [17] found learners generally responded well to interprofessional education, with their knowledge, attitudes, and skills improved. An essential premise of interprofessional education is that if students learn together, they can learn from each other and be better prepared for IPCP and teamwork, which can ultimately enhance care for the clients and their families [18].

Some respondents proposed simulation-based teaching when delivering interprofessional education. It has been suggested that simulation helps expose students to clinical situations that are unlikely to be experienced in the classroom [19]. The use of scenarios adapted from real case conferences encourages learning through informal discussion among students about the simulated clients’ conditions and functions of other health professionals, and stimulates their critical thinking capacity [20]. The use of scenarios in simulation-based teaching in interprofessional education also enhances students’ compassion and empathetic communication with the clients and their relatives [21].

In addition to education, organisational support is necessary to promote IPCP. Several respondents in this study expressed concerns about liability issues in delivering care to older adults. Liability affects interprofessional collaboration as team members have broader and overlapping scopes of practice [22]. This may require regulatory bodies and health care institutions revisiting and revising established legal frameworks to enable the interprofessional team to function in a coordinated manner and maintain safe care.

Our study has several limitations. First, we interviewed a purposive sample of 16 aged care providers. Although the sample constituted members representing most professions involved in aged care, inclusion of more allied health professionals such as dieticians and podiatrists to understand their views of and roles in IPCP would be beneficial to generalise the results. Second, this was a qualitative study and a quantitative perspective would bolster the findings through analysis of the importance of IPCP, its associated factors, and the impact of interprofessional education on IPCP. Third, most respondents had over 10 years of work experience. Studies are needed to explore the perceptions of junior, less experienced health professionals and how they advance their knowledge and skills in IPCP to better inform strategies to prepare them for IPCP.

## Conclusion

The study highlights the importance of IPCP in providing person-centred coordinated care for older adults across various healthcare settings. The practice of IPCP required shared decision making among the collaborating health professions. To promote IPCP, interprofessional team members need to understand and respect each other’s professional roles and scope of practice. Engaging in effective communication and acknowledging concerns over liabilities are also important. Offering interprofessional education early in each health professional’s degree programme and adopting innovative simulation-based teaching strategies are likely to promotie IPCP and enhance health outcome of people at old age. These findings will inform the development of interprofessional gerontological education to further enhance students’ competence in IPCP.

## Data Availability

The datasets used and/or analysed during the current study are available from the corresponding author on reasonable request.

## Abbreviations

BScG: Bachelor of Science in Gerontology Programme
IPCP: Interprofessional Collaborative Practice
IPE: Interprofessional Education
